# Estimating on the fly: The approximate number system in rufous hummingbirds (*Selasphorus rufus*)

**DOI:** 10.3758/s13420-020-00448-z

**Published:** 2020-12-14

**Authors:** Mia Corliss, Theo Brown, T. Andrew Hurly, Susan D. Healy, Maria C. Tello-Ramos

**Affiliations:** 1 School of Biology, University of St Andrews, St Andrews, KY16 9TH UK; 2 Department of Biological Sciences, University of Lethbridge, Lethbridge, Alberta Canada

**Keywords:** Approximate number system, Foraging, Numerosity, Rufous hummingbird

## Abstract

When presented with resources that differ in quantity, many animals use a numerosity system to discriminate between them. One taxonomically widespread system is the approximate number system. This is a numerosity system that allows the rapid evaluation of the number of objects in a group and which is regulated by Weber’s Law. Here we investigated whether wild, free-living rufous hummingbirds (*Selasphorus rufus*) possess an approximate number system. The hummingbirds were presented with two experiments. In the first we investigated whether hummingbirds spontaneously chose an array containing more flowers than an alternate array. In the second we asked whether the hummingbirds could learn to use numerosity as a cue to which of two arrays contained the better reward. The birds did not spontaneously prefer an array containing more flowers. After minimal training, however, they learned to choose the more numerous array and could differentiate between arrays of five and seven flowers. These data support the presence of an approximate number system in the rufous hummingbird. It seems plausible that having such a system would enable much more efficient foraging in this species.

**Supplementary Information:**

The online version contains supplementary material available at 10.3758/s13420-020-00448-z.

## Introduction

When it comes to survival, the name of the game is efficiency. Organisms live and die by how much energy they expend and acquire during their daily lives (Hurly, 2003). The more energy that is saved, the more energy can be dedicated towards reproduction. A simple way to gain energy is to exploit only the most valuable resource available, and one way to determine the value of a resource is by its numerosity.

There are three main systems employed to evaluate numerosity: the object-tracking system with highly accurate and rapid evaluation of small numbers; counting, with highly accurate but slow evaluation of large numbers; and the approximate number system with rapid but low accuracy evaluation of large numbers (Carey, 2009; Pepperberg, 2017). Each of these systems is best suited to different tasks, dependent on which system’s associated shortcomings carry the smallest impact in terms of fitness. The object-tracking system is useful when assessing small quantities (e.g. the difference between one or two peanuts), the counting system is useful if the time spent is outweighed by the cost of being wrong (e.g. a bird not detecting an extra egg laid by a brood parasite in its nest), while the approximate number system is useful when the cost of picking the lesser resource is outweighed by the time needed to count and discriminate between resources (e.g. a solitary zebra hesitating between joining a herd of 100 or 110 zebra and losing the protection of either).

Animals appear to use the approximate number system to rapidly determine the numerosity of groups that are too large for the object tracking system (N > 3–4) (Feigenson & Carey, 2005; Hyde, 2011). This system allows the production of rough counts of resources, such as mates, predators or food, and is found in a wide variety of species: beetles (*Tenebrio molitor*), various fish species (*Gambusia holbrooki*, *Gambusia affinis*), red-backed salamanders (*Plethodon cinereus*), some birds (*Petroica longipes*, *Corvus corone*, *Psittacus erithacus*) and multiple primates (Agrillo, Dadda, Serena, & Bisazza, 2008; Beran, 2007; Carazo, Font, Forteza-Behrendt, & Desfilis, 2009; Dadda, Piffer, Agrillo, & Bisazza, 2009; Ditz & Nieder, 2016; Garland, Low, & Burns, 2012; Hanus & Call, 2007; Pepperberg, 1994; Uller, Jaeger, Guidry, & Martin, 2003). While this system provides an approximation and not an exact value, it is far more rapid and requires less effort and attention than the exact alternative of counting. The details of this approximation can be summarised using Weber’s Law: the just-noticeable difference between two stimuli is proportional to the size of the stimuli, rather than remaining a constant amount. In the context of the approximate number system, this means that the just-noticeable difference between two numerosities changes as a constant ratio as the numerosities change (Cantlon & Brannon, 2006; Hauser, Tsao, Garcia, & Spelke, 2003), or that the approximate number system becomes more imprecise as the ratio between the values approaches 1.

Rufous hummingbirds (*Selasphorus rufus*) are very small vertebrates (weighing around 3–4 g; Chai & Millard, 1997) and are fiercely territorial (Carpenter, Hixon, Temeles, Russell, & Paton, 1993). Feeding as they do almost exclusively on flower nectar (López-Calleja, Fernàndez, & Bozinovic, 2003), their high metabolic rate and expensive flight leads to them living on tight energy budgets. The foraging ecology of these birds appears to have favoured a variety of cognitive abilities: they can learn and track the reward refill rates of the flowers that they visit (Henderson, Hurly, Bateson, & Healy, 2006; Tello-Ramos, Hurly, Higgott, & Healy, 2015b) and produce traplines, where they repeatedly use only a small selection of possible routes between flowers (which are often also the most efficient routes; Tello-Ramos, Hurly, & Healy, 2015a, 2019).

Rufous hummingbirds might thus be expected to have other methods of saving energy, such as using systems that allow them to distinguish between resources of different quality by the cues presented, as already seen with spatial location and colour (Hurly & Healy, 2002). And, given that these birds can correctly locate a single rewarded flower in a sequential array of ten flowers, using a numerical ability called ordinality (Vámos, Tello-Ramos, Hurly, & Healy, 2020), then it seems plausible they might use an approximate number system to allow them to choose foraging opportunities based on the number of flowers available. If these birds have an approximate number system then, when multiple options (e.g. plants) are available, we would expect them to choose to visit first plants or patches that offer more rewarded resources or to defend locations that have more flowers available. We tested this possibility by offering wild, free-living rufous hummingbirds a series of pairs of patches of artificial flowers, in which each of the pairs contained a different number of flowers. We expected the birds to visit first the patch that contained more flowers.

## Methods

**Fig. 1:**
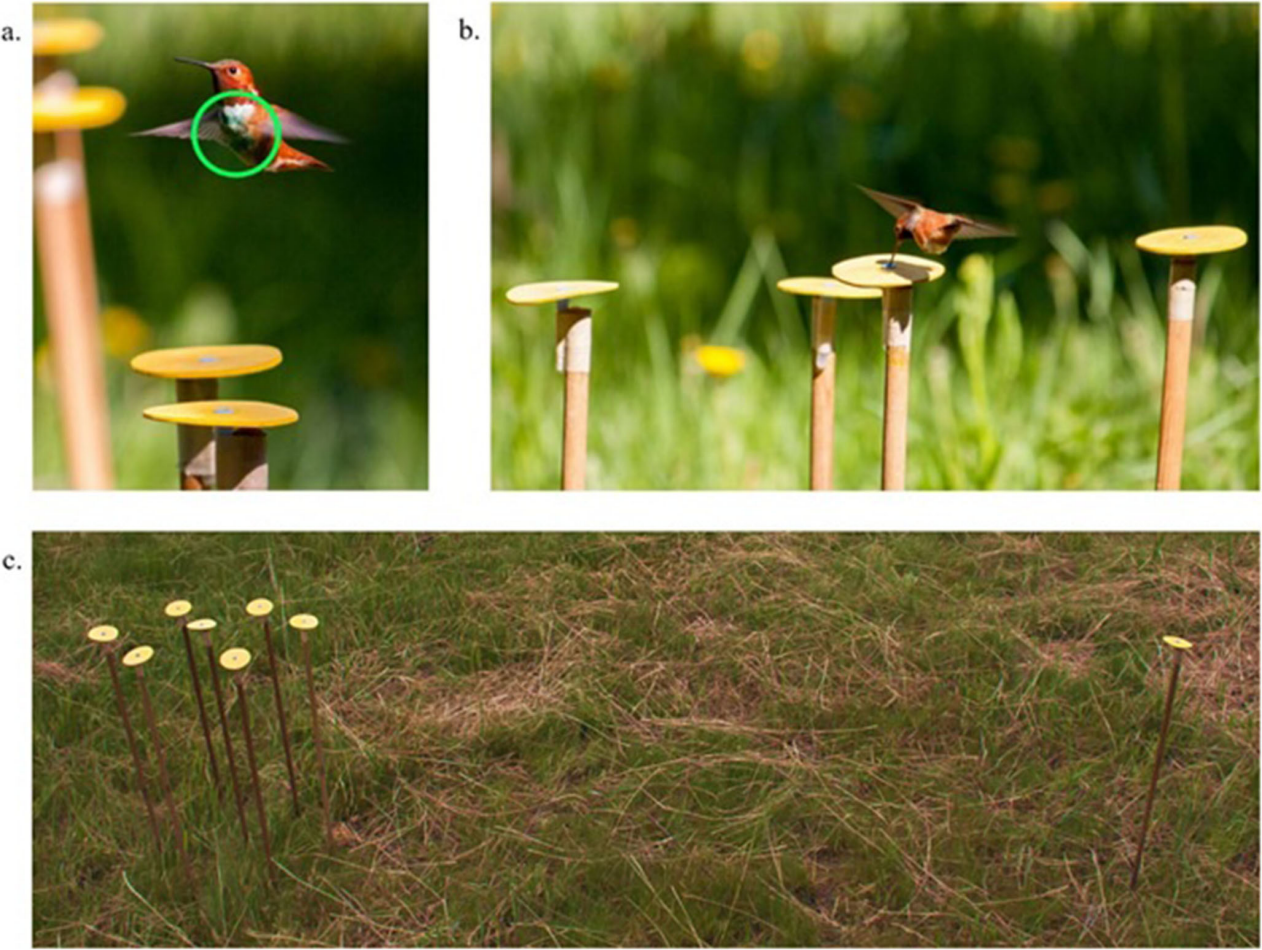
Photographs of the subjects and the tests. **a** A male rufous hummingbird hovers above a test array made up of artificial flowers. The circle surrounds the marking used to identify individual birds. **b** A male rufous hummingbird feeding from a flower in a training array, which contains four rewarded flowers. The training array in the first experiment was arranged in a square shape. **c** Example of a test layout. Here the test depicted is a choice between seven flowers and one flower.

The subjects were wild male rufous hummingbirds in Westcastle Valley (N49.349153, W114.410864), Southern Alberta in the Canadian Rocky Mountains. Male rufous hummingbirds established individual territories around individual commercial hummingbird feeders containing a 16% w/w sucrose solution at sites along the valley in early May. Once a male had been identified as territorial (consistently feeding from and defending the feeder from intruders), he was captured and marked on the feathers on his upper chest and back using a non-toxic water-based ink (Jiffy Eco-marker Ink) to allow individual identification (Fig. 1a).

One day after a bird was marked, we trained him to feed from an experimental flower containing a 25% w/w sucrose solution. The artificial flowers (hereafter referred to as “flowers”) were made of a yellow foam sheet cut into a circle with a 6 cm diameter (Fig. 1b). A hole in the middle of the flower held a 120 μL syringe tip with the needle removed, which held the sucrose. The flowers (made from the yellow plastic-foam circles and a syringe tip) were supported by syringe caps taped to 60-cm long sticks stuck in the ground. The syringe caps acted as holders for the flowers, keeping the flowers stable (Fig. 1b). Male rufous hummingbirds are readily trained (within 1 or 2 h) to feed from different types of artificial flowers not least because these birds preferentially use spatial over visual information when learning to feed from a new resource (Tello-Ramos et al., 2014). Depending on the type of experiment, however, these birds will use the most salient cue when learning which flowers are rewarded and which are not (Healy & Hurly, 2013). For the current study, all flowers were visually identical and during flower training we reduced the possibility that the birds used spatial cues to learn which flower contained reward by moving the training flower at least 1 m away from the previous location between feeding bouts. Once a bird was flower trained the experiments began.

## Ethical note

The University of St Andrews Ethics Committee and the University of Lethbridge Animal Welfare Committee approved all work, which was also conducted under permit from the Alberta Sustainable Resource Development and Environment Canada.

## Experimental design

### Experiment 1: Do hummingbirds prefer an array with more flowers?

The subjects were 13 wild male rufous hummingbirds. The experiment consisted of a series of training trials and tests. A visit was defined as a bird having fed from at least one flower in the array and leaving the array for at least 30 s.

During training we removed the bird’s feeder and presented him with a square array of four flowers, with each flower 20 cm from its two nearest neighbours (Fig. 1b). Each flower contained 25 μL of 25% sucrose solution, leading to a total of 100 μL available for consumption on each visit. We presented the training array for five visits, moving the array 50–100 cm between each visit so that birds would associate the reward with the flowers rather than with the flowers’ spatial locations. Between visits, we replaced all flowers with other, identical flowers and refilled with the same sucrose volume as before. The replacement flowers were haphazardly selected from a bag and used to reduce the possibility of associating the presence or absence of sucrose with visual cues of a particular flower (Hornsby et al., 2014).

**Fig. 2:**
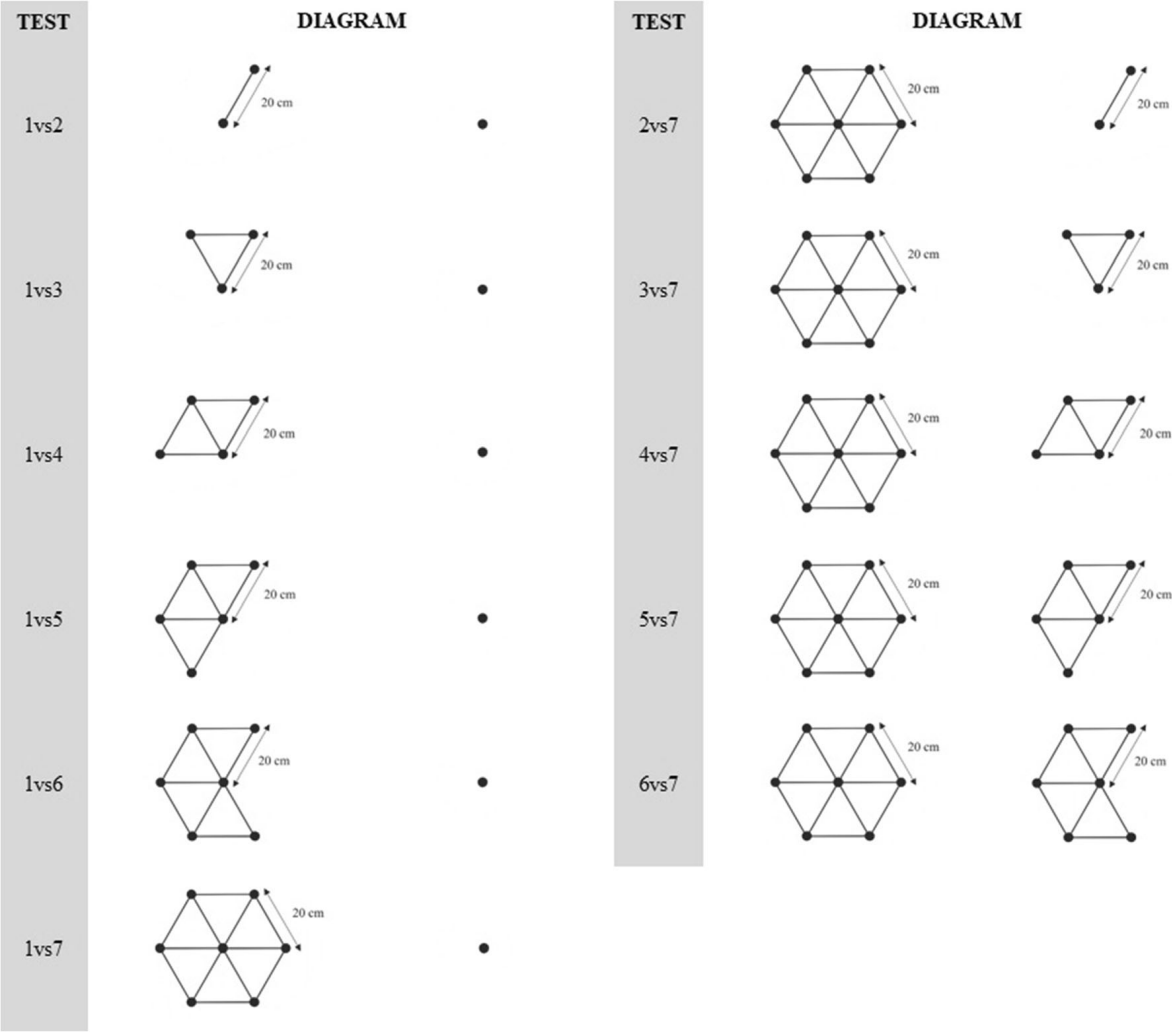
Illustrations of the 11 test layouts presented to the experimental subjects. Dots represent the placement of the experimental flowers. Lines represent the spacing between the flowers, set at 20 cm. The centre point of each of the two arrays was 2 meters from the other.

Once the bird had visited the training array five times, we presented the first probe test array. Each test was composed of two flower arrays that were 2 m apart, set so that the location of the last training array before the tests was at the midpoint between the two test arrays. All flowers in the test arrays were empty. The order of training and testing was repeated so that the bird was presented with a test after every set of five training visits (e.g., five training trials, test 1, five training trials, test 2, etc.). This was done to reduce the probability that the birds learned that flowers were not rewarded when they were presented with the pairs of arrays. In total there were 11 tests with five training visits between each test. The first test consisted of one array of seven flowers and one array of one flower (Fig. 1c). The second test consisted of one array of six flowers and one array of one flower. This pattern continued until the sixth test, which was one array of two flowers and one array of one flower (e.g. one vs. seven, one vs. six, one vs. five, and so on). The seventh test consisted of one array of seven flowers and one array of two flowers. The eighth test presented one array of seven flowers and one array of three flowers (e.g., two vs. seven, three vs. seven, four vs. seven, and so on). This pattern continued until the 11th test, which was one array of six flowers and one array of seven flowers (Fig. 2).

When the hummingbird visited the test array, we recorded which of the two arrays the bird visited first. We alternated the position of the more numerous array with each test from left to right, where ‘left’ and ‘right’ were defined as relative to the experimenter’s location when looking towards the feeder location. The angle of the test layout was then fixed for all tests at this site from this point and did not rotate relative to the feeder.

The next training array was then set up 50–100 cm from the location of the last training array, and the training trials and tests continued from there.

### Experiment 2: Can hummingbirds learn to use numerosity to choose the array containing a reward?

The subjects were nine wild male rufous hummingbirds. All nine birds had already completed Experiment 1 and started the second experiment between 10 and 22 days of completing the first. As all birds were experienced with feeding from the artificial flowers, we did not need to train them again. Eight birds completed both sets of the tests, whereas one bird completed only one set of tests (one vs*.* two through one vs*.* seven but not two vs*.* seven through six vs*.* seven).

The experiment consisted of two training trials and two sets of test trials, the same 11 tests as in Experiment 1 but this time the flowers in each array were rewarded and the reward was different, depending on the number of flowers in each array. During training we first presented an array of seven flowers, each containing 25 μL of 25% sucrose solution. Once the bird had visited this array once, we presented a single flower array in which the flower contained 25 μL of 5% sucrose solution, a sucrose concentration that is much less preferred by the hummingbirds (Morgan, Hurly, & Healy, 2014). The test trials then began.

The test trials were made up of two arrays set 2 m apart. One array was made up of flowers that held 25 μL of 25% sucrose solution, while the other array was made up of flowers that held 25 μL of 5% sucrose solution. The array in which the flowers contained 25% sucrose always had more flowers than did the 5% array, and we pseudorandomly changed the position of the array with flowers containing 25% sucrose.

As in Experiment 1, in each test we recorded which array the bird visited first. The first set of tests went as follows. The first test consisted of one array of one flower and one array of seven flowers (i.e. Test 1 = one vs. seven). We used one of two criteria to end a test. The first criterion was reached if the bird visited the array with more flowers first three times in a row. The second criterion was completing ten visits to the pair of arrays. Once one of the criteria was fulfilled, the test ended and the next test began. For the next test in this set we presented one array of one flower and one array of six flowers. This pattern continued until the sixth test, which was one array of two flowers and one array of one flower (i.e., Test 2 = one vs. six, Test 3 = one vs. five, and so on; Fig. 2).

The second set of tests went as follows. In the first test we presented the bird with one array of two flowers and one array of seven flowers, in the second test we presented one array of three flowers and one array of seven flowers (i.e., two vs*.* seven, three vs. seven, four vs. seven, and so on). This pattern continued until the fifth test, which was one array of six flowers and one array of seven flowers (Fig. 2).

Of the eight birds that completed all the tests, four received the first set of tests first and four received the second set of tests first. Once all tests were completed, the feeder was returned to its original location.

## Data analysis

We used one-sample t-tests to determine whether the birds visited the larger array significantly more often than would be expected by chance (50%) at the 0.05 significance level. For Experiment 1, where each subject was tested with each test condition only once, we use binomial tests to determine if more birds visited the larger (more numerous) array more often than expected by chance. We used Spearman correlation tests to determine whether choices to the larger array (the percentage of birds in Experiment 1; percentage of choices in Experiment 2) was correlated with either the total number of flowers present during a test or with the ratio of the number of flowers between the two arrays. We used a t-test to compare the number of times birds visited the more numerous array when the constant array had one flower than when it had seven flowers. We used one-sample t-tests to determine if the birds visited the array with more flowers for each test in Experiment 2. We used Spearman correlation tests to determine if the performance of birds during Experiment 1 correlated with the performance of birds in Experiment 2. We used a two-sample t-test to compare the percentage of correct choices by each bird between Experiment 1 and Experiment 2. Where appropriate we report effect size as *r* for t-tests and Cohen’s d in the case of the binomial tests.

## Results

### Experiment 1: Do hummingbirds prefer an array with more flowers?

**Fig. 3:**
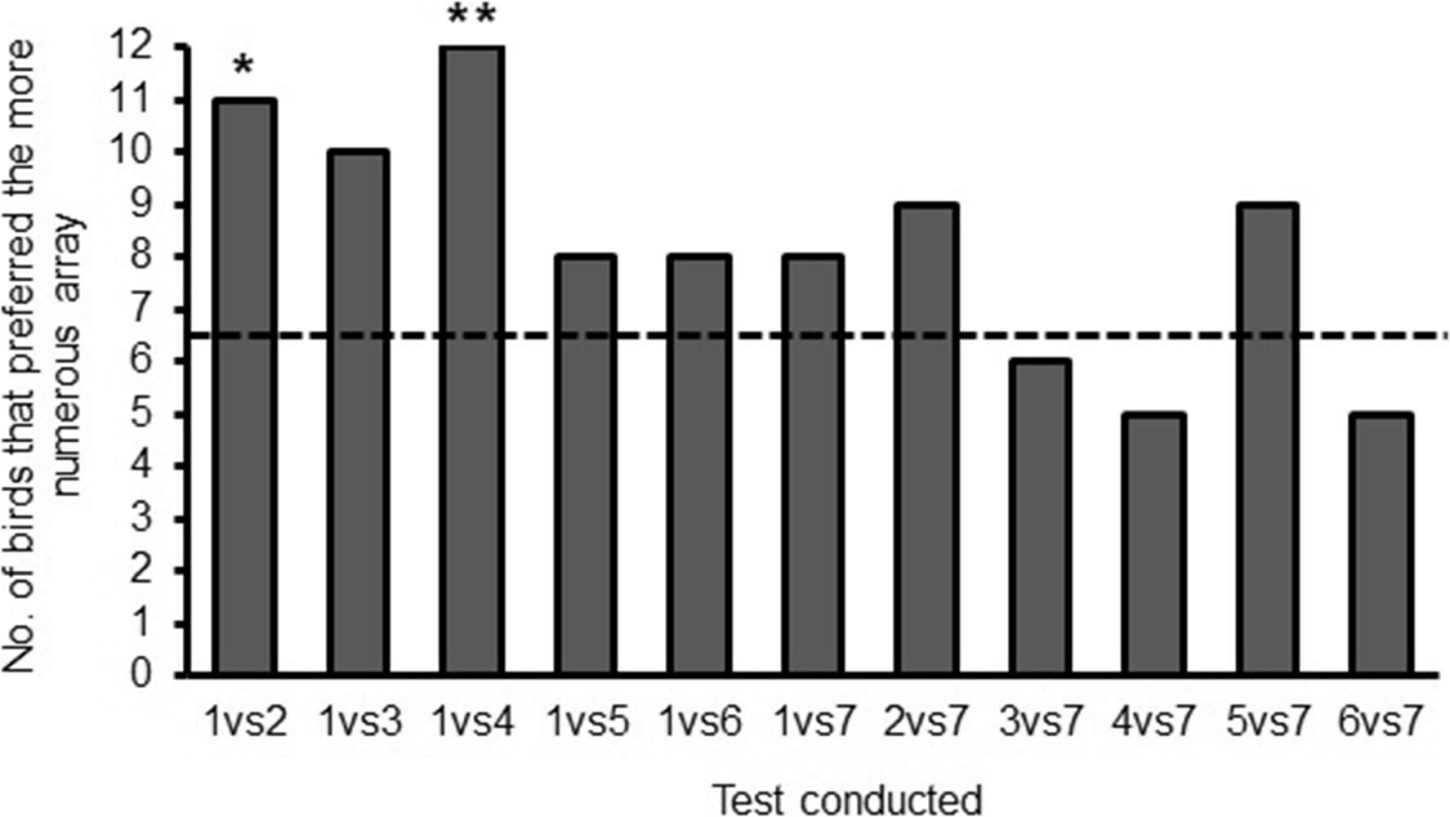
The number of birds in each test that visited the array containing more flowers in Experiment 1 for each of the pairs of arrays. The dashed line indicates the expected performance if the birds performed according to chance (50%). Asterisks indicate performance that was statistically significant from chance: ** p < 0.01, *p < 0.05.

Thirteen birds completed this experiment. On average across all tests, birds visited the more numerous array significantly more than expected by 50% chance (mean ± SE: 63.63 ± 3.71; t_12_ = 3.67, p < 0.01, *r* = 0.72). Examination of choices relative to each test condition (Fig. 3) similarly reveals an overall greater number of choices to the larger array, but for only two of the test conditions were the results significant (one vs. two (ratio 0.5): 11/13, Z = 2.50, p = 0.023, Cohen’s d = 0.74; one vs. four (ratio 0.25): 12/13, Z = 3.05, p = 0.003, Cohen’s d = 0.99 ; see Table 1 in Online Supplementary Material for other outcomes). No test conditions resulted in the smaller array being chosen significantly more often than chance.

**Fig. 4:**
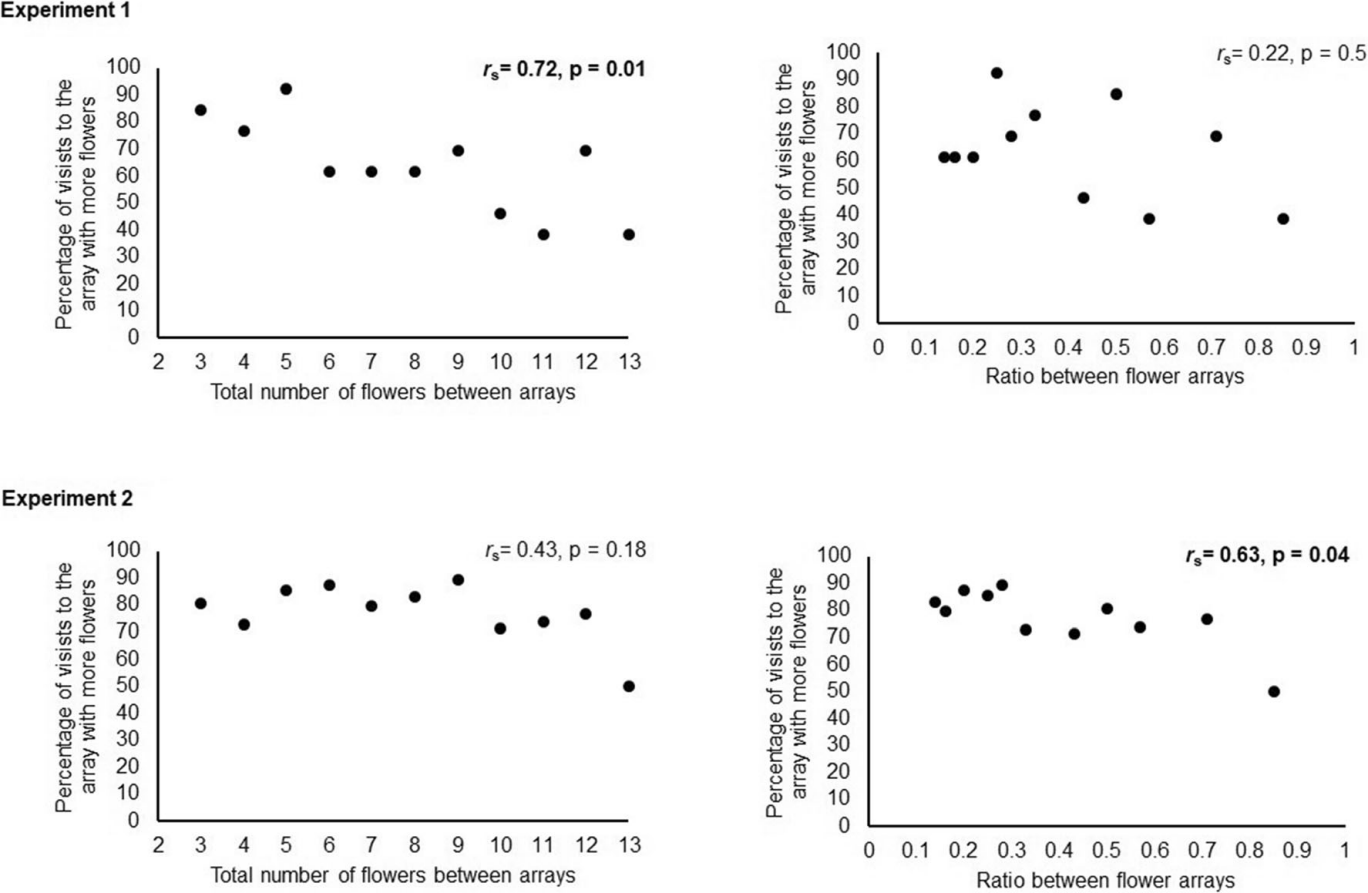
Correlations between the number of choices to the more numerous array and test condition, when test condition was characterized either by the total number of flowers (sum of the two arrays) or the ratio of the two arrays (small/large).

We performed correlation analysis to determine whether some aspects of test conditions influenced choices. The number of birds choosing the larger array was negatively correlated with the total number of flowers in the two arrays (*r*_*s*_ *=* 0.72, p = 0.01, n = 13; Fig. 4). The ratio of the array sizes (small/large) had no significant effect on choices (*r*_s_ = 0.22, p= 0.5, n = 13; Fig. 4).

An additional way to examine the effect of the number of flowers on the birds’ choices is to divide the tests into two separate series: those in which one of the arrays always contains only one flower versus those in which one of the arrays always contains seven flowers (Figs. 2 and 3). We excluded the test condition *one versus seven* because it was not unique to one of these series. Birds visited the array with more flowers significantly more frequently when the constant array was the array with one flower (75.38 ± 4.02) than when the constant array had seven flowers (52.31 ± 3.60; t_12 =_ 6.04, p < 0.001*, r* = 0. 86).

### Experiment 2: Can hummingbirds learn to use numerosity to choose the array containing a reward?

**Fig. 5:**
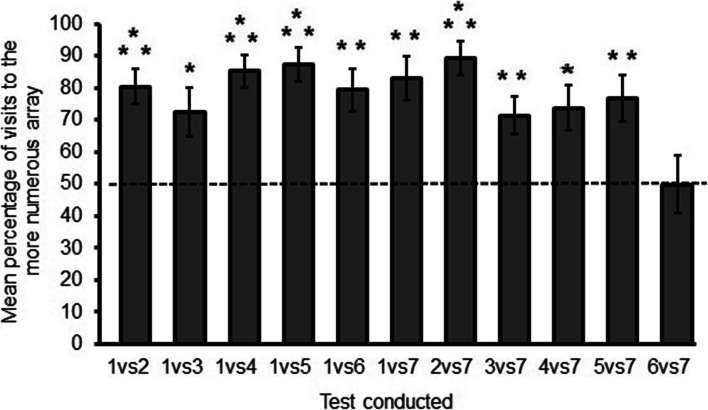
The proportion of visits to the array containing more flower for each of the pairs of arrays. The dashed line indicates the expected performance if the birds performed according to chance (50%). Asterisks indicate performance that was statistically significant: *** p < 0.001, ** p < 0.01, * p < 0.05.

Hummingbirds visited the array with more flowers significantly more than expected by 50% chance (mean ± SE: 77.54 ± 1.62; t_8_ = 16.97, p < 0.001, *r* = 0.98). In all of the tests except one (six vs. seven, ratio = 0.85: t_7_ = 0.007, p = 0.99, *r* = 0.003), birds visited the array that had more flowers significantly more than expected by 50% chance (Fig. 5; all t_7/8_ ≥ 2.9, all p ≤ 0.01, all *r* ≥ 0.72 ; see Online Supplementary Material, Table 2).

As in Experiment 1, test condition did influence choices, but in this case the ratio of the number of flowers between arrays was more important than the total number of flowers in the test. The percentage of visits to the larger array increased as the relative size of the arrays (smaller ratio) increased (*r*_s_ = 0.63, p = 0.04, n = 9; Fig. 4). Choices were not correlated with the total number of flowers in the test (r_s_ = 0.43, p = 0.18, n = 9; Fig. 4). There was, however, an effect of flower number when we compared all test conditions in which one of the arrays always contained one flower (81.93 ± 1.87) versus test conditions in which one of the arrays always contained seven flowers (71 ± 2.74; t_7 =_ 3.04, p = 0.01*, r* = 0.75).

Choices to the more numerous array were significantly greater in Experiment 2 (77.54 ± 1.62) than in Experiment 1 (63.63 ± 3.71; t_20_ = 2.22 p = 0.03, *r* = 0.44). To examine whether the two experiments assessed similar aspects of numerosity, we correlated the mean responses of the nine birds common to the two experiments but found no significant relationship (r_s_ = 0.26, p = 0.49, n = 9).

## Discussion

In Experiment 1, in which birds were trained to feed from an array of four, equally rewarded, flowers and then tested with pairs of flower arrays containing different numbers of flowers (all empty), overall the birds spontaneously chose the array containing more flowers. However, detailed examination of each test condition revealed limited significant choices. In Experiment 2, we specifically trained the birds that the larger training array contained a higher quality reward than the smaller array, and tests indicated that they recognized all but one of the differences in array size. Detailed analyses revealed that choices to the larger array were influenced by the total number of flowers in Experiment 1, and by the relative size of the two arrays (ratio) in Experiment 2.

The data from both experiments are consistent, but the data from Experiment 1 are weaker than those from Experiment 2. These latter demonstrate that until the two arrays contained seven and six flowers each (ratio = 0.85), the birds could distinguish between them, and chose to visit the more numerous array. The difference in outcome between the two experiments is not especially surprising, as with Experiment 1 we tapped into spontaneous choices, whereas in Experiment 2 we both explicitly linked numerosity to resource quality and provided some minor training. In this context the birds not only were able to choose the more appropriate array, they did so readily up until a ratio of the numbers of flowers in the two arrays consistent (or better than) with the ratios that other animals are capable of discriminating. For example, mosquitofish (Agrillo et al., 2008), guppies (*Poecilia reticulata*) (Lucon-Xiccato, Petrazzini, Agrillo, & Bisazza, 2015), and New Zealand robins can all discriminate up to ratios of 1:2 (Garland et al., 2012); chicks (*Gallus domesticus*) can discriminate one versus two (ratio = 0.5) and two versus three (ratio = 0.66) stimulus sets but not sets of four versus six, four versus five, and three versus four (ratios = 0.6, 0.8, and 0.75, respectively; Rugani, Regolin, & Vallortigara, 2008; Rugani, Vallortigara, & Regolin, 2013b); rhesus macaques (*Macaca mulatta*) approached whichever of two boxes had the larger quantity of apple pieces when the differences in pieces was one versus two, two versus three, three versus four, and three versus five (ratios = 0.5, 0.66, and 0.6, respectively; Hauser, Carey, & Hauser, 2000), while apes will discriminate between a pile of nine pellets and another of ten pellets (ratio = 0.9; Hanus & Call, 2007), albeit with much greater training than our hummingbirds experienced. Honey bees (*Apis mellifera*) can not only discriminate between similar ratios, including zero versus one (Howard et al.*,* 2018), but they can also spontaneously transfer their choices to match size rather than number (Bortot, Stancher, & Vallortigara, 2020).

That our hummingbirds provided evidence of their numerical discrimination much more strongly in Experiment 2 is consistent with data from bees: in both cases, when the animals were trained that one of the options contained the reward and the other did not (Howard et al., 2019), both bees and our hummingbirds showed that they were capable of numerical discrimination that is not seen when the training was much less, and importantly, involved only a rewarding option (Howard et al.*,* 2020). Indeed, substantial differences in the numerical abilities of animals are found depending on whether spontaneous numerical abilities are tested or if animals are first trained to associate a reward with a higher or lower numerosity (Agrillo & Bisazza, 2014). Our hummingbirds appeared to show a spontaneous preference for the more numerous array only when the choice was between one versus two and one versus four, but as the training array was made up of four flowers, it is possible that the preference for the more numerous array in the latter choice was due to the similarity to the training array rather than to spontaneous discrimination.

Given that the hummingbirds do appear to possess an approximate number system (see also Vámos et al., 2020), their lack of spontaneous discrimination suggests that they do not use the system to preferentially visit a more numerous resource over another less numerous resource when they are naïve to the quality of both resources. Although this behaviour might at first sight seem inefficient, it is consistent when considered in the context of the rufous hummingbird’s ecology. The nectar in the flowers that the birds visit across the day can vary in both quality and quantity depending on whether the flowers have already been visited (e.g. Healy & Hurly, 1995; Tello-Ramos, Hurly, Higgott, et al., 2015b), the age of the flowers (Pleasants, 1983), the ambient temperature (Jakobsen & Kritjánsson, 1994), and even the time of day (Pleasants, 1983). Furthermore, while some plants produce many flowers, others produce few or just one, often with more or richer nectar. In the normal foraging situations of these hummingbirds, then, numerosity is far from being a guarantee of resource quality. A hummingbird that has experienced variation in quantity and quality of flowers very probably should sample flowers without paying much attention to their numerosity. But as has been shown for so many instances with rufous hummingbirds, these birds use different types of cues based on their reliability for the task at hand. For example, these birds will typically use spatial cues over colour cues ( Hurly, Franz, & Healy, 2010; Tello-Ramos, Hurly, & Healy, 2014), but not always (Hornsby, Hurly, Hamilton, Pritchard, & Healy, 2014), or may use colour cues to potentiate learning about time (Marshall, Hurly, Sturgeon, Shuker, & Healy, 2013; Samuels, Hurly, & Healy, 2014), but not always (Marshall, Hurly, & Healy, 2012).

In summary, wild, free-living rufous hummingbirds are able to discriminate between arrays of flowers based on the number of flowers in those arrays, which is consistent with these birds having an approximate number system. These hummingbirds may not be able to match Alex’s ability to give voice to his numeracy (e.g. Pepperberg, 1987; Pepperberg & Carey, 2012), but coupled with the evidence that they also can also determine ordinality in a sequence of flowers, they provide evidence that ecology may have shaped numerical capabilities.

## Supplementary Information

ESM 1 (DOCX 16 kb)

ESM 2 (DOCX 16 kb)
