# Supplementary material for: Estimating on the fly: The approximate number system in rufous hummingbirds (Selasphorus rufus)
Source: Learn Behav. 2020 Dec 14;49(1):67–75. doi: 10.3758/s13420-020-00448-z (PMC7979633; doi:10.3758/s13420-020-00448-z)
Supplement: Supplementary file 1 — (DOCX 16 kb) [file 13420_2020_448_MOESM1_ESM.docx]

Table 2

| **Test** | **Ratio** | **Total** | **N** | **Percentage of visits** | **t** | **p-value** | **Effect size** |
| --- | --- | --- | --- | --- | --- | --- | --- |
|  |  | **flowers** |  | **to numerous array** |  |  |  |
| 1vs.2 | 0.5 | 3 | 9 | 80.5 | 5.61 | **< 0.001** | 0.89 |
| 1vs.3 | 0.33 | 4 | 9 | 72.6 | 2.95 | **< 0.05** | 0.72 |
| 1vs.4 | 0.25 | 5 | 9 | 85.3 | 7.06 | **< 0.001** | 0.92 |
| 1vs.5 | 0.2 | 6 | 9 | 87.4 | 6.95 | **< 0.001** | 0.92 |
| 1vs.6 | 0.16 | 7 | 9 | 79.4 | 4.4 | **< 0.01** | 0.84 |
| 1vs.7 | 0.14 | 8 | 9 | 83.1 | 4.77 | **< 0.001** | 0.86 |
| 2vs.7 | 0.28 | 9 | 8 | 89.4 | 7.55 | **< 0.001** | 0.94 |
| 3vs.7 | 0.43 | 10 | 8 | 71.5 | 3.71 | **< 0.01** | 0.81 |
| 4vs.7 | 0.57 | 11 | 8 | 73.7 | 3.35 | **< 0.05** | 0.78 |
| 5vs.7 | 0.71 | 12 | 8 | 76.8 | 3.7 | **< 0.01** | 0.81 |
| 6vs.7 | 0.85 | 13 | 8 | 49.9 | 0.007 | 0.99 | 0.003 |
