# Supplementary material for: Estimating on the fly: The approximate number system in rufous hummingbirds (Selasphorus rufus)
Source: Learn Behav. 2020 Dec 14;49(1):67–75. doi: 10.3758/s13420-020-00448-z (PMC7979633; doi:10.3758/s13420-020-00448-z)
Supplement: Supplementary file 2 — (DOCX 16 kb) [file 13420_2020_448_MOESM2_ESM.docx]

Table 1

| **Test** | **Ratio** | **Total** | **N** | **No. of birds to more numerous array** | **Percentage of visits to numerous array** | **Z-score** | **p-value** | **Effect size** |
| --- | --- | --- | --- | --- | --- | --- | --- | --- |
|  |  | **flowers** |  |  |  |  |  |  |
| 1vs.2 | 0.5 | 3 | 13 | 11 | 0.85 | 2.5 | **0.023** | 0.76 |
| 1vs.3 | 0.33 | 4 | 13 | 10 | 0.77 | 1.94 | 0.092 | 0.56 |
| 1vs.4 | 0.25 | 5 | 13 | 12 | 0.92 | 3.05 | **0.003** | 1 |
| 1vs.5 | 0.2 | 6 | 13 | 8 | 0.62 | 0.83 | 0.581 | 0.23 |
| 1vs.6 | 0.17 | 7 | 13 | 8 | 0.62 | 0.83 | 0.581 | 0.23 |
| 1vs.7 | 0.14 | 8 | 13 | 8 | 0.62 | 0.83 | 0.581 | 0.23 |
| 2vs.7 | 0.29 | 9 | 13 | 9 | 0.69 | 1.39 | 0.133 | 0.39 |
| 3vs.7 | 0.43 | 10 | 13 | 6 | 0.46 | -0.28 | 0.71 | 0.07 |
| 4vs.7 | 0.57 | 11 | 13 | 5 | 0.38 | -0.83 | 0.867 | 0.23 |
| 5vs.7 | 0.71 | 12 | 13 | 9 | 0.69 | 1.39 | 0.133 | 0.39 |
| 6vs.7 | 0.85 | 13 | 13 | 5 | 0.38 | -0.83 | 0.867 | 0.23 |
